# Prevalence of Imposter Syndrome and Its Risk Factors Among University of Sharjah Medical Students

**DOI:** 10.7759/cureus.57039

**Published:** 2024-03-27

**Authors:** Zinab Alzufari, Rosul Makkiyah, Aisha Alowais, Aisha Almazrouei, Abdul Kareem A Abu Ali, Abdulaziz Alnaqbi, Jibran Sualeh Muhammad

**Affiliations:** 1 College of Medicine, University of Sharjah, Sharjah, ARE; 2 Department of Basic Medical Sciences, College of Medicine, University of Sharjah, Sharjah, ARE

**Keywords:** psychology health, psychiatry, united arab emirates (uae), medical education, occupational health, behavioral health, medical students, imposter syndrome

## Abstract

Background

Imposter syndrome describes an internal experience of intellectual fraud, where individuals attribute their academic or occupational endeavors and achievements primarily to luck rather than to their diligent efforts. Additionally, the stringent standards and prerequisites set by medical institutions create an environment conducive to impostorism among medical students. This study aimed to evaluate the prevalence and severity of imposter syndrome among medical students at the University of Sharjah.

Methodology

This research was designed as a descriptive cross-sectional study. A total of 400 participants enrolled in the study using non-probability convenience sampling, but 399 participants, 49.4% (197) from colleges of medicine and 50.6% (202) from dentistry, successfully completed the questionnaire. Participants completed a questionnaire containing the Clance Imposter Phenomenon Scale. Statistical associations between variables were tested using the chi-square test. Individuals with chronic medical conditions or those using medications with known psychiatric side effects were excluded.

Results

The analyzed sample comprised 399 students, with 64.7% females and 35.3% males. Most respondents were from year 2 (21.3%, 85), while the fewest were from year 5 (18.3%, 73). The majority of students fell into the categories of moderate (46.4%, 185) and frequent (35.8%, 143) imposter experiences. Among all investigated characteristics, pure academic factors such as field of study (p = 0.001), study phases (p = 0.032), advisor’s attitude (p = 0.029), and comparison with peers’ performance and grades (p = 0.024 and <0.001, respectively) exhibited the highest significant association with the severity of imposter syndrome.

Conclusions

This study revealed a high prevalence of imposter syndrome among medical students, emphasizing the need for comprehensive strategies and interventions targeting academically associated risk factors to alleviate the burden of imposter syndrome.

## Introduction

The term imposter syndrome was first used by Clance and Imes (1978) [1] to describe an internal experience of intellectual fraud that seemed to be prevalent among high-achieving women [2]. The term was later modified by Harvey [3] to define a failure to internalize success and viewing oneself as an impostor which included any individual facing achievement tasks regardless of their success, status, or gender [4,5]. Amid the competitive nature of medical schools and their requirements for high-achieving individuals, medical schools can be a breeding environment for impostorism among medical students.

This can further be denoted in a study that showed the prevalence of the imposter phenomenon in nearly half of medical student participants [6]. However, the strive for perfectionism and the continued series of self-doubt does not end after medical school. In a study conducted among healthcare professionals, imposter syndrome was found to be fairly common, occurring at least once in the career of 70% of the participants included in the study [4,7].

Although impostor syndrome has not been identified as a mental illness as per the Diagnostic and Statistical Manual of Mental Disorders or International Classification of Diseases, it is an experience that significantly impacts a person’s psychological well-being and could extend its effects to physical and professional damage [4]. In a study correlating levels of burnout, its indices, and the presence of impostor syndrome among American medical students, statistically significant associations were established [6]. Burnout components that were found to be greatly associated with the experience of impostor syndrome included exhaustion, emotional exhaustion, cynicism, and depersonalization [6]. Those disturbing results intensify the fact that acknowledging such a psychological experience and raising awareness about it can help young healthcare individuals overcome the hardships of the initial stages of their careers.

Physicians suffering from impostor syndrome attribute their success and qualifications to factors other than hard work; consequently, they become unable to internalize a sense of competence and praiseworthy [6]. Moreover, impostor syndrome was found to be significantly associated with burnout indices [8-10]. With individuals adopting prolonged workaholic behaviors to achieve unrealistic goals, a vicious cycle of recrimination, exhaustion, and burnout [11] that impedes the student’s performance in medical school is created, further exacerbating these feelings.

To date, there are no research studies from the United Arab Emirates or the Arab region to understand this common phenomenon. Despite impostor syndrome being a very common phenomenon among healthcare students, according to the literature mentioned earlier, the impact of real or imagined underperformance on seemingly competent healthcare students is poorly understood in the region and preventive measures are yet to be taken. Therefore, the purpose of this study is to develop a deeper understanding of impostor syndrome by evaluating its prevalence among medical and dental students. Moreover, determining the stages at which impostorism may emerge and acknowledging the factors that may contribute to impostorism can aid in establishing strategies aimed at supporting medical students who struggle with imposter syndrome.

## Materials and methods

Study design

This study was designed as a descriptive, quantitative, cross-sectional study as the outcome of this study was to be measured in a descriptive, observational approach at one moment in time. The research proposal was approved by the University of Sharjah Research Ethics Committee (approval number: REC-20-01-27-04-S). The study was conducted at the University of Sharjah, United Arab Emirates, from January 2020 until October 2020.

Sample and sampling

Sampling was done via a non-probability convenience approach. Inclusion criteria included all five levels of core medical and dental programs to identify our study population. Students from the College of Medicine levels 1-5 and from the College of Dentistry levels 1-5 were eligible to be included in the study. The purpose and method of the study were explained to the participants. It was ensured that their participation was voluntary, anonymous, and confidential. Because our study focused on assessing the psychological aspects of impostorism and its manifestations, undergraduates with known chronic medical illnesses or those who were taking drugs that could cause any psychological side effects were excluded. The following formula was used to calculate a sample size of 399 participants: (n = 4 p (1 - p)/SE2), where n is the required sample size and p is the expected prevalence % (50), and the margin of error was set at 5%.

The data collection was physical and occurred over two months, starting from January 2020 and extending until the end of February 2020. The questionnaire was printed (400 copies) and then distributed among the group members. A group member approached students during breaks or at the end of classes and explained the study goals and the anonymity of participation. In addition, an information sheet was printed with each copy and was offered to participants for additional information. After verbal consent was obtained from participants, the group member remained available to supervise the questionnaire-filling process and in case the participants had any additional questions or doubts regarding the questions. Once the participants had filled out the questionnaires, the group member collected them. This method of collection was chosen to minimize bias and ensure participants were filling in the responses with full subjectivity.

Instruments and techniques

The introduction sheet informed that the identification of participant responses would be anonymous in the provided data and would be utilized solely for this study. The self-administered questionnaire used in this study included three main sections, namely, demographics, environment (family, birth order, peers, academic advisors), and the Clance Imposter Phenomenon Scale (CIPS) that was developed by Pauline Rose Clance [12] (Appendix). The first section included questions about demographics such as gender, age, college program, academic level, and volunteer work. The second section assessed each individual’s environment between his/her family, peers, academic advisors, and external social influence. Individual’s environment among family members was assessed by asking about birth order, the presence of medical professionals in the family, and the influence of family members on one’s choice of his/her major. Regarding the environment between his/her peers, the individual’s performance was compared with their colleagues and peers. Moreover, we enquired about the students’ experiences with academic advising and whether the advisors had a positive or negative impact on their academic performance. Finally, we further assessed the influence of social media on the students. The third section included the CIPS, a structured psychometric instrument, that was used to assess the severity of imposter feelings among medical students by identifying feelings of self-doubt, failure to repeat success, and fear of being less capable than others [12]. The 20-item questionnaire, each consisting of a five-point scale, was used to determine the severity of impostorism from none to very severe (1-5), yielding a cumulative score ranging from 20 to 100. If the total score was 40 or less, the respondent had few impostor characteristics; if the score was between 41 and 60, the respondent had moderate imposter experiences; a score between 61 and 80 implied that the respondent frequently had impostor feelings; and a score higher than 80 implied that the respondent often had intense imposter experiences.

Statistical analysis

Questionnaire data were collected and stratified according to the study program (medicine or dentistry) and academic level (years 1-5). Furthermore, data were entered manually and analyzed using SPSS version 25 (IBM Corp., Armonk, NY, USA). Descriptive statistics (mean, standard deviation, frequency, and percentage) were used. Pearson’s chi-square test was used to study the association between external factors correlating with the impostor phenomenon. The statistical level of significance was set at a p-value ≤0.05.

## Results

Out of 400 students enrolled in the study, 399 returned the questionnaires, with a response rate of 99.75%. Male students (141) accounted for 35.3% of the participants, while female students (258) accounted for 64.7%. The mean age of the participants in this study was 20.97 years, except for one outlier which was 27. Table 1 shows the demographic characteristics of the participants.

**Table 1:** Participants’ demographics and academic demographics.

Participants’ demographics and academic demographics	Respondents (N = 399)
Gender	
Female	64.7% (258)
Male	35.3% (141)
Age (years)	
<21	44.2% (175)
21–27	55.8% (221)
Field of study	
Medicine	49.4% (197)
Dentistry	50.6% (202)
Level of study	
Preclinical (years 1 and 2)	40.9% (163)
Transition (year 3)	21.1% (84)
Clinical (years 4 and 5)	38.1% (152)

Family environment

Demographically, 95.2% (380) of the study participants had siblings while a small minority had no siblings. The largest number of respondents were middleborns at 42.4% (162), followed by firstborns at 38.7% (148), and, finally, lastborns at 18.8% (72) of the population. The majority of participants (69.7%, 278) lived with their families while only 13% (52) lived alone. The other 17.3% (69) lived with either a roommate or a friend. Additionally, participants were asked if their place of residence affected their self-confidence. Almost half (46.1%, 184) reported that their confidence increased, while 45.9% (183) reported no effect. A minority of the participants (7%, 28) reported lower confidence due to their place of residence.

Level and field of study

Concerning response rate, there was a symmetrical distribution. Medical students (197) represented 49.4% of the respondents, while dentistry students (202) constituted 50.6%. The response rates obtained from each academic year were relatively similar between the two majors. The highest response rate was from year 2 students at 21.3% (85), whereas year 5 students had the lowest response rate at 18.3% (73). More than half of the participants (56.4%, 225) had a family member in the medical field while 43.6% (174) did not. In addition, 82.9% (329) of the participants said that they were influenced to choose the major they are currently pursuing, while only a small minority chose their major on their own accord.

Peers’ performance and grades

Academically, most of the undergraduates (44.6%, 178) believed that their peers were more hard-working than them. This percentage was relatively close to those who reported no difference in their performance levels. Overall, 52.6% (210) of the respondents did not share their feelings about their performance with others whereas the rest of them felt comfortable enough to share it. Half of the participants (54.6%, 218) reported relatively similar grades to their peers while 16% (64) reported higher and 14.8% (59) received lower grades.

Impact of advisor’s attitude

Less than half of the participants (46.9%, 187) thought that an advisor’s attitude had an impact on their academic performance. Out of this category, 71.2% (133) thought that the impact was positive on their academic performance.

Volunteer work and impact on students’ confidence level

Substantially, 65.4% (261) of the respondents took part in volunteer work. Among them, 82.0% (214) became more self-confident whereas 17.2% (45) did not feel any change in their self-confidence after volunteering.

Imposter syndrome severity

**Figure 1:**
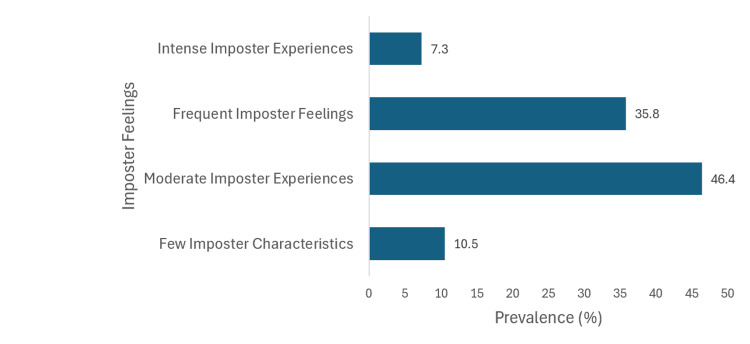
Severity of impostor syndrome.

Participants were asked to answer a series of 20 questions, with each question scaled from 1 to 5, where a score of 1 referred to “not true at all” and “5” to “very true,” for each question. Then, the cumulative scores were compared to the Clance Imposter Scale. Figure 1 shows the pattern of severity of impostor syndrome.

The results depicted a high prevalence and severity of imposter syndrome in students at the colleges of medicine and dentistry, with the majority of participants (89.5%, 357) experiencing moderate, frequent, or intense imposter syndrome characteristics. According to the Clance scale, almost half of the respondents (46.4%, 185) were in the “moderate imposter experiences” category while 35.8% (143) had frequent imposter feelings, and less than 10% (29) experienced intense imposter experiences; additionally, 10.5% (42) experienced few imposter feelings. Table 2 shows the association between all variables and the severity of impostor syndrome.

**Table 2:** Bivariate analyses of the severity of imposter syndrome in association with various factors (N = 399). *: Significant at a p-value ≤0.05.

Variables	Total	Few imposter characteristics	Moderate imposter experiences	Frequent imposter feelings	Intense imposter experiences	Chi-square p-value
Demographics						
Age (years)						
<21	44.2% (175)	8.6% (15)	47.4% (83)	34.9% (61)	9.1% (16)	0.351
21–27	55.8% (221)	12.2% (27)	45.7% (101)	36.7% (81)	5.4% (12)	
Gender						
Male	35.3% (141)	9.2% (13)	51.8% (73)	33.3% (47)	5.7% (8)	0.412
Female	64.7% (258)	11.2% (29)	43.4% (112)	37.2% (96)	8.1% (21)	
Academic demographics						
Field of study						
Medicine	49.4% (197)	9.1% (18)	44.7% (88)	33.5% (66)	12.7% (25)	0.001*
Dentistry	50.6% (202)	11.9% (24)	48.0% (97)	38.1% (77)	2.0% (4)	
Level of study						
Preclinical (years 1 and 2)	40.9% (163)	9.8% (16)	47.2% (77)	36.8% (60)	6.1% (10)	0.032*
Transition (year 3)	21.1% (84)	10.7% (9)	35.7% (30)	38.1% (32)	15.5% (13)	
Clinical (years 4 and 5)	38.1% (152)	11.2% (17)	51.3% (78)	33.6% (51)	3.9% (6)	
Academical environment						
Advisor’s attitude experience						
Positive	71.2% (131)	9.9% (13)	49.6% (65)	31.3% (41)	9.2% (12)	0.029*
Negative	28.8% (53)	3.8% (2)	32.1% (17)	47.2% (25)	17.0% (9)	
Peers performance						
More hardworking	44.6% (178)	8.4% (15)	39.9% (71)	40.4% (72)	11.2% (20)	0.024*
No difference	43.9% (175)	12.6% (22)	53.1% (93)	30.9% (54)	3.4% (6)	
Less hardworking	11.5% (46)	10.9% (5)	45.7% (21)	37.0% (17)	6.5% (3)	
Peers’ grades						
Higher	18.8% (64)	12.5% (8)	51.6% (33)	32.8% (21)	3.1% (2)	0.000*
Relatively close	63.9% (218)	10.6% (23)	52.3% (114)	32.1% (70)	5.0% (11)	
Lower	17.3% (59)	5.1% (3)	27.1% (16)	47.5% (28)	20.3% (12)	
Comparison and influence						
Volition in the choice of major						
Yes	82.9% (329)	11.2% (37)	47.1% (155)	34.7% (114)	7.0% (23)	0.381
No	17.1% (68)	5.9% (4)	42.6% (29)	42.6% (29)	8.8% (6)	
Precedence of the medical profession in family						
Yes	56.4% (225)	13.3% (30)	43.6% (98)	35.1% (79)	8.0% (18)	0.158
No	43.6% (174)	6.9% (12)	50.0% (87)	36.8% (64)	6.3% (11)	
Order of birth						
Firstborn	38.7% (148)	11.5% (17)	44.6% (66)	38.5% (57)	5.4% (8)	0.254
Middleborn	42.4% (162)	11.1% (18)	50.0% (81)	32.7% (53)	6.2% (10)	
Lastborn	18.8% (72)	6.9% (5)	43.1% (31)	36.1% (26)	13.9% (10)	
Effect of public figures						
Positively	74.5% (161)	11.2% (18)	46.6% (75)	34.8% (56)	7.5% (12)	0.541
No influence	25.5% (55)	5.5% (3)	54.5% (30)	34.5% (19)	5.5% (3)	
Dependency						
Accommodation status						
Alone	13.0% (52)	5.8% (3)	50.0% (26)	38.5% (20)	5.8% (3)	0.612
With family	69.7% (278)	11.5% (32)	47.1% (131)	34.9% (97)	6.5% (18)	
With someone	17.3% (69)	10.1% (7)	40.6% (28)	37.7% (26)	11.6% (8)	
Volunteer work						
Yes	65.9% (261)	11.1% (29)	47.9% (125)	34.9% (91)	6.1% (16)	0.585
No	34.1% (135)	9.6% (13)	43.0% (58)	38.5% (52)	8.9% (12)	

The variables that showed significant associations are described below.

Field of Study

**Figure 2:**
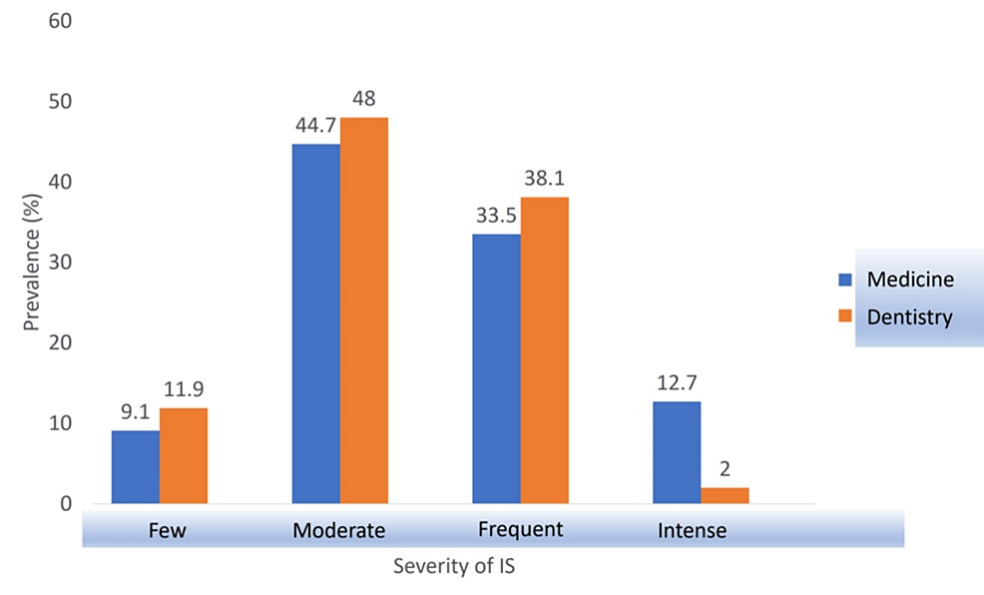
Association between IS severity and the field of study. IS: imposter syndrome

With the p-value (0.001) being lower than the significance level (0.05), the alternative hypothesis was accepted, meaning that the field of study showed a significant effect on the severity of imposter syndrome. As demonstrated in Figure 2, dental students were more concentrated on the lower end of the CIPS scale, with a total of 121 students showing either moderate or few imposter syndrome characteristics in contrast to the 106 medical students. However, medical students were found to be rather concentrated on the higher end of the imposter scale, with more students showing intense imposter characteristics. Moreover, 12.7% (25) of medical students experienced intense imposter feelings compared to only 2% (4) of dental students.

Study Phase

**Figure 3:**
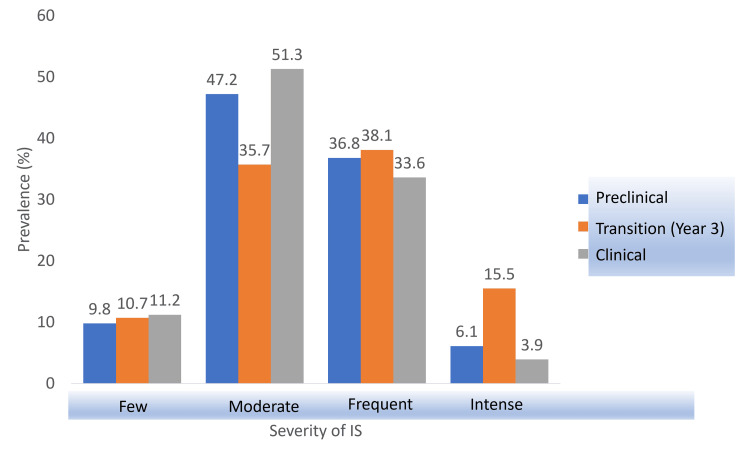
Association between IS severity and study phases. IS: imposter syndrome

After dividing the sample according to the academic phase, the p-value was found to be 0.032, which was lower than the significance level (0.05). Therefore, the alternate hypothesis indicating the correlation between the different phases of study and the severity of imposter syndrome among medical students was accepted. Figure 3 shows that students in preclinical years, along with the transitional phase (year 3), showed more severe levels of imposter syndrome than clinical years; both preclinical and transitional phases included a larger number of students suffering from frequent and intense imposter feelings in contrast to the clinical stage which constituted mainly of students experiencing only few and moderate imposter characteristics. More importantly, however, is the fact that transitional year students (year 3) had the highest percentage of students suffering from imposter syndrome falling on the higher end of the CIPS scale, with 15.5% (13) of year 3 students experiencing intense imposter feelings in contrast to only 6.1% (10) and 3.9% (6) of preclinical (years 1 and 2) and clinical (years 4 and 5) students, respectively.

Advisor’s Attitude

**Figure 4:**
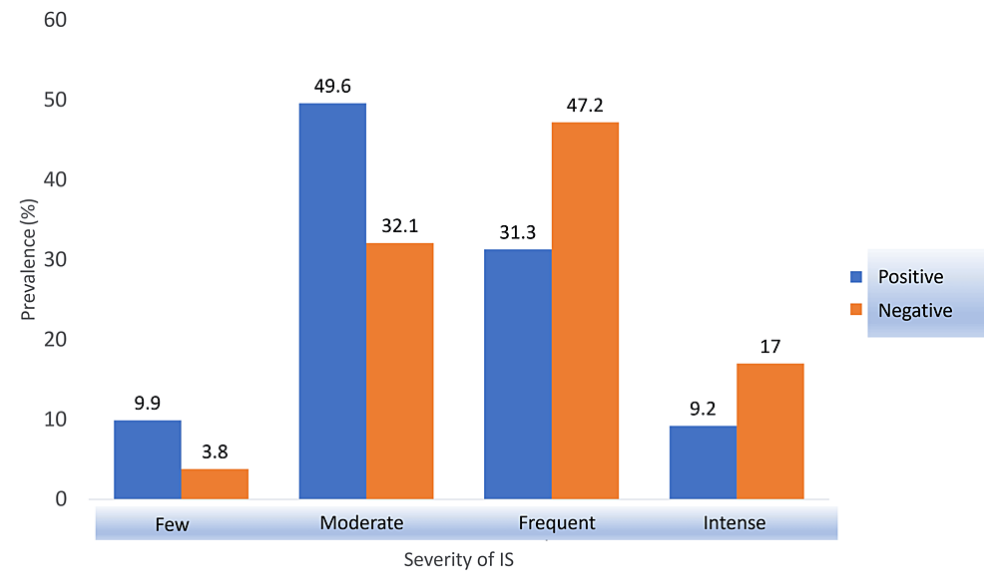
Association between IS severity and advisor’s attitude. IS: imposter syndrome

Those who reported that they had encountered an advisor whose attitude affected them were asked about the nature of the impact. Figure 4 demonstrates that 28.8% of those 187 participants reported a negative experience due to a certain attitude from an advisor, and the prevalence of frequent and intense imposter feelings in those participants was 15.9% and 7.8% higher than the other group (with a positive experience), respectively. With a p-value of 0.029, which was lower than the significance level (0.05), the null hypothesis was rejected, and the alternate hypothesis indicating the correlation between an advisor’s attitude and the severity of imposter syndrome among medical students was accepted.

Peers’ effect

Peers’ Performance

**Figure 5:**
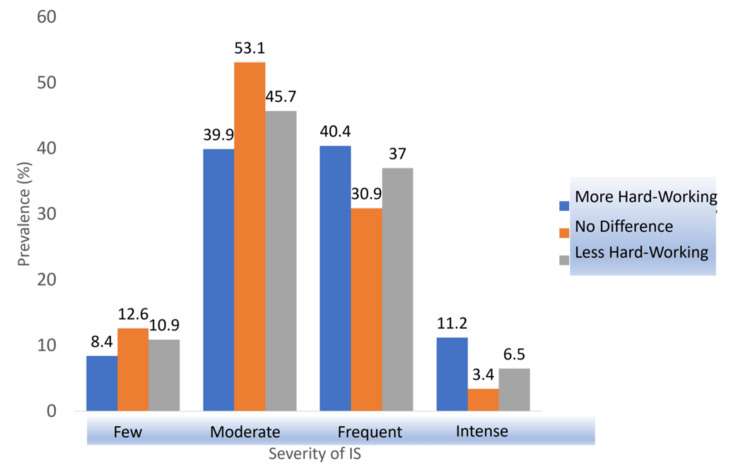
Association between IS severity and peers’ performance. IS: imposter syndrome

Peers’ performance (p = 0.024) was reported as either being more hardworking, less hardworking, or not different from the respondent. As shown in Figure 5, 11.2% (20) of those who believed that their peers were more hardworking experienced intense imposter feelings compared to only 3.4% (6) and 6.5% (11) from the other two groups, who reported that their peers are equally or even less hard-working, respectively. Furthermore, the prevalence of frequent imposter feelings in the first group was significantly high, with differences of 9.5% between them and the second “no difference” group and 3.4% between them and the third “less hardworking” group. Hence, the null hypothesis was rejected.

Peers’ Grades

**Figure 6:**
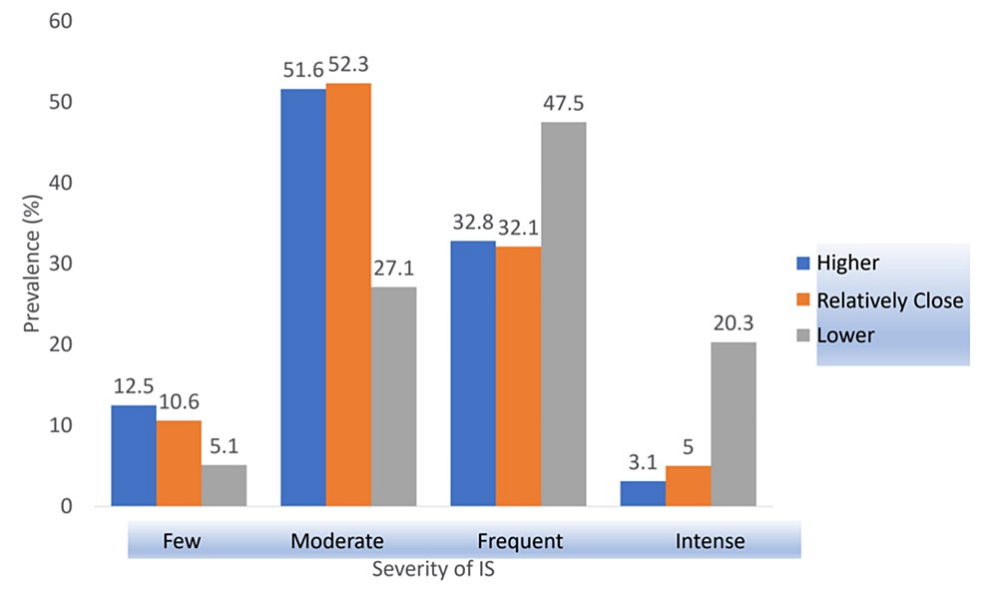
Association between peers’ grades and IS severity. IS: imposter syndrome

As for peers’ grades, a highly significant association was shown by the p-value (0.000), which was greatly lower than 0.05. Figure 6 shows that the group of participants who reported having lower grades than their peers had a higher prevalence of frequent and intense imposter feelings with a difference of at least 15.3% and 14.7% compared to those having relatively close or higher grades than their peers, respectively.

Age and Gender

When investigating the association between age and the severity of imposter syndrome, the p-values were found to be 0.351 and 0.412, respectively, both of which were higher than the significance level (0.05). Thus, the null hypothesis for both variables was accepted. The rest of the following factors had a p-value higher than the level of significance (0.05) which deemed the null hypothesis true for all the following factors. When comparing the results of those who chose their major out of their own will to those who were influenced, the frequency and intensity of imposter experiences showed minor differences (p = 0.381). Furthermore, whether participants had any family members in the medical field or not did not show any significant association with imposter feelings (p = 0.158). Furthermore, the order of birth showed a p-value of 0.254, and the influence of public figures in the field of medicine or dentistry on the students showed a p-value of 0.541, both of which were higher than the significance level (0.05). Finally, in this study, dependent characteristics such as accommodation and volunteer work had no significant p-values.

## Discussion

The primary goal of this study was to reveal the prevalence and severity of imposter syndrome among medical and dental students, which can help set starting points for intervention measures. The results depicted a high prevalence and severity of imposter syndrome in students at the colleges of medicine and dentistry with the majority of participants (89.5%, 357) experiencing moderate, frequent, or intense imposter syndrome characteristics. These findings align with those of previous studies which denoted that the highest percentages of students were in the category of moderate and frequent imposter syndrome severity [12]. Such results are alarming and must be taken into consideration as the severity of moderate and intense imposter syndrome shows that this syndrome is significant and is not restricted to general or few imposter syndrome characteristics. The variables that have significant associations with the severity of imposter syndrome were purely academic in this study. These results highlight the importance of directing interventions toward academic factors to limit this rising phenomenon.

According to this study’s results, non-academic factors showed no marked influence on the development of imposter feelings. For instance, in line with recent studies, this study showed no significant association between gender and the severity of imposter feelings [13]. This contrasts previous definitions of imposter syndrome and early studies, where imposter syndrome was thought to be a condition solely among high-achieving females and was reflected in many subsequent early studies to be higher in females [14-17]. This finding establishes evidence for altered patterns of impostor severities among males and provides new insights into the prevalence of such conditions in both genders.

Contrary to our expectations, being the first in the family to enter the fields of medicine or dentistry had no significant association with the prevalence and severity of imposter syndrome. The results contradicted those of Canning et al. [18], which correlated being a first-generation student with having a significantly higher risk of developing imposter syndrome characteristics. Other non-academic factors that had no significant association with the severity of the imposter phenomenon were being experienced in volunteer work and whether a student was living on their own. This nullifies the hypothesized association between independence and imposter syndrome.

One of the academic factors that made the participants prone to feelings of imposter syndrome was the advisor’s attitude toward the student. Experiencing a positive attitude appeared to have a marked influence on the severity of imposter syndrome. This showed the importance of focusing on ensuring a comfortable environment for advising. In our study, students who had a positive experience were more likely to be at the lower end of the scale of imposter syndrome. This finding builds on an existing one on the effects of perceived competition and the development of imposter traits [18].

The perception of peers’ grades had the highest significance in association with the severity, proving that comparing results and achievements with colleagues had a significant influence on the development of imposter syndrome feelings. As such a syndrome depends on comparing abilities and knowledge with others, our hypothesis on the significance of this factor’s impact was in line with the results.

In correlation with previous studies, students in the transitional stages, from preclinical to clinical, had the highest prevalence of intense imposter syndrome feelings, indicating that such stages expose students to enough stress and accelerate the development of severe imposter syndrome characteristics [19]. In addition, the difference between the proportion of students who reported having intense imposter syndrome characteristics was greater in medicine than in dentistry, showing an increased likelihood of higher stress and competition. These results place great emphasis on the surge of imposter syndrome indices, especially in transitional stages.

Limitations

Limitations to note in this study involve self-reported data through questionnaires which can introduce inaccurate self-judgment and scores. In addition, the collection of data from one single medical school with the current sample size can prevent generalizability. Additionally, having a larger number of medical students would help reveal more significant associations and raise the level of accuracy.

Strengths and implications

A strength of this study is that the group members supervised data collection to prevent any misunderstanding of the questions by the participants and ensure accurate judgment before answering. The use of a validated scale and the exploration of various demographic and environmental factors is also an added strength of the study. The results reveal significant facts about the factors mostly associated with impostor syndrome. These factors being academic will allow authorities in medical schools to take action in enhancing the psychological aspects of the journey. Additionally, the nature of the associated factors found in this study can help medical schools implement curricular or extracurricular strategies to raise awareness and help students suffering from impostor feelings overcome them. Future studies can extend the exploration of other associated factors such as burnout syndrome or psychological distress, which are possible coexisting conditions to be investigated [13,15].

## Conclusions

This study assessed the prevalence and severity of impostor characteristics among medical students and showed that the majority had moderate features. Students at the College of Medicine, especially those in their transitional year, were shown to have a higher prevalence of severe impostor feelings, highlighting a tendency of increased self-doubt and burnout at this stage. Most that were significantly associated with the severity of imposter syndrome in this study were academic, highlighting the necessity for future interventions to limit this condition. More studies should be performed to assess the extent of this condition among healthcare workers and its effects on the delivery of care. Discovering what significantly affects imposter syndrome development and its severity can be beneficial in the prevention and management of this syndrome in medical individuals.

Acknowledging the factors that are most likely to hasten the development and progression of imposter syndrome can help establish efficient intervention campaigns and programs. Not only does imposter syndrome affect the present of medical individuals but it can also hinder any future advancements and future productivity. Therefore, early actions to minimize such experiences can help reduce added burdens. Encompassing factors with their significant associations can aid in taking future actions that would improve both the academic and non-academic experience in medical school. Furthermore, exploring other contributing factors early can lead to a much better understanding of different aspects of this condition and would likely help in the early identification of imposter syndrome in the medical field.

## Appendices

**Table 3:** Imposter syndrome prevalence and severity questionnaire.

Section A. Demographics and environment	Answers	
1. What is your sex?	a. Male	
	b. Female	
2. How old are you? (in years)	Please specify: _________	
		3. Do you have any siblings?	a. Yes	
b. No (skip to the next question)		
3a. What birth order are you?	a. Firstborn	
	b. Middleborn	
	c. Lastborn	
4. Do you live:	a. Alone	
	b. With family	
	c. With someone (friends/dormmate, etc.)	
5. How do you think your place of residence affects your self-confidence?	a. Increased confidence	
	b. Decreased confidence	
	c. No change	
6. What field are you currently enrolled in	a. Medicine	
	b. Dentistry	
7. At present, which academic year are you in?	a. Year 1	
	b. Year 2	
	c. Year 3	
	d. Year 4	
	e. Year 5	
8. Did you choose your major? (select No if someone influenced you to select your major)	a. Yes	
	b. No	
9. Other than yourself, is anyone in your family pursuing a career in the field of medicine, such as a (physician, dentist, nurse, pharmacist, lab technician, etc.)?	a. Yes	
	b. No	
10. Compared to yourself, do you feel your peers are:	a. More hardworking	
	b. No difference	
	c. Less hardworking	
10a. How would you describe your grades in comparison to theirs?	a. Higher	
	b. Relatively close	
	c. Lower	
	d. I don’t know	
	11. When you feel bad regarding a specific academic performance you are most likely to:	a. Keep it to yourself	
b. Share it with others		
12. Have you noticed that an advisor’s attitude toward you has had an impact on your academic conduct?	a. Yes	
	b. No (skip to the next question)	
12a. If yes, was it a positive or a negative impact?	a. Positive	
	b. Negative	
12b. Do you believe that having a better approach from your advisor would improve your academic conduct?	a. Yes	
	b. No	
13. Do you follow any public figure/social media influencer who is a medical/dental student?	a. Yes	
	b. No (skip to the next question)	
13a. How would you say this person influenced you?	a. Positively	
	b. No influence	
	c. Negatively	
14. Have you ever done any volunteer work?	a. Yes	
	b. No (skip to the next question)	
14a. If yes, how do you think volunteering affected your self-confidence?	a. I became more self-confident	
	b. I became less self-confident	
	c. I don’t feel any change in my self-confidence	
Section B. Clance Imposter Phenomenon Scale	Answers	
1. I have often succeeded in a test or task even though I was afraid that I would not do well before I undertook the task	a. Not at all	
	b. Rarely	
	c. Sometimes	
	d. Often	
	e. Very true	
2. I can give the impression that I’m more competent than I really am	a. Not at all	
	b. Rarely	
	c. Sometimes	
	d. Often	
	e. Very true	
3. I avoid evaluations if possible and have a dread of others evaluating me	a. Not at all	
	b. Rarely	
	c. Sometimes	
	d. Often	
	e. Very true	
4. When people praise me for something I’ve accomplished, I’m afraid I won’t be able to live up to their expectations of me in the future	a. Not at all	
	b. Rarely	
	c. Sometimes	
	d. Often	
	e. Very true	
5. I sometimes think I obtained my present position or gained my present success because I happened to be in the right place at the right time or knew the right people	a. Not at all	
	b. Rarely	
	c. Sometimes	
	d. Often	
	e. Very true	
6. I’m afraid people important to me may find out that I’m not as capable as they think I am	a. Not at all	
	b. Rarely	
	c. Sometimes	
	d. Often	
	e. Very true	
7. I tend to remember the incidents in which I have not done my best more than those times I have done my best	a. Not at all	
	b. Rarely	
	c. Sometimes	
	d. Often	
	e. Very true	
8. I rarely do a project or task as well as I’d like to do it	a. Not at all	
	b. Rarely	
	c. Sometimes	
	d. Often	
	e. Very true	
9. Sometimes I feel or believe that my success in my life or in my job has been the result of some kind of error.	a. Not at all	
	b. Rarely	
	c. Sometimes	
	d. Often	
	e. Very true	
10. It’s hard for me to accept compliments or praise about my intelligence or accomplishments	a. Not at all	
	b. Rarely	
	c. Sometimes	
	d. Often	
	e. Very true	
11. At times, I feel my success has been due to some kind of luck	a. Not at all	
	b. Rarely	
	c. Sometimes	
	d. Often	
	e. Very true	
12. I’m disappointed at times in my present accomplishments and think I should have accomplished much more	a. Not at all	
	b. Rarely	
	c. Sometimes	
	d. Often	
	e. Very true	
13. Sometimes I’m afraid others will discover how much knowledge or ability I really lack	a. Not at all	
	b. Rarely	
	c. Sometimes	
	d. Often	
	e. Very true	
14. I’m often afraid that I may fail at a new assignment or undertaking even though I generally do well at what I attempt	a. Not at all	
	b. Rarely	
	c. Sometimes	
	d. Often	
	e. Very true	
15. When I’ve succeeded at something and received recognition for my accomplishments, I have doubts that I can keep repeating that success	a. Not at all	
	b. Rarely	
	c. Sometimes	
	d. Often	
	e. Very true	
16. If I receive a great deal of praise and recognition for something I’ve accomplished, I tend to discount the importance of what I’ve done	a. Not at all	
	b. Rarely	
	c. Sometimes	
	d. Often	
	e. Very true	
17. I often compare my ability to those around me and think they may be more intelligent than I am	a. Not at all	
	b. Rarely	
	c. Sometimes	
	d. Often	
	e. Very true	
18. I often worry about not succeeding with a project or examination, even though others around me have considerable confidence that I will do well	a. Not at all	
	b. Rarely	
	c. Sometimes	
	d. Often	
	e. Very true	
19. If I’m going to receive a promotion or gain recognition of some kind, I hesitate to tell others until it is an accomplished fact	a. Not at all	
	b. Rarely	
	c. Sometimes	
	d. Often	
	e. Very true	
20. I feel bad and discouraged if I’m not “the best” or at least “very special” in situations that involve achievement	a. Not at all	
	b. Rarely	
	c. Sometimes	
	d. Often	
	e. Very true	
